# Combination of Platelet-Rich Plasma and Hyaluronic Acid vs. Platelet-Rich Plasma Alone for Treatment of Knee Osteoarthritis

**DOI:** 10.3390/biomedicines11102759

**Published:** 2023-10-12

**Authors:** Ashim Gupta, Surya Prakash Sharma, Anish G. Potty

**Affiliations:** 1 Regenerative Orthopaedics, Noida 201301, India; 2 Future Biologics, Lawrenceville, GA 30043, USA; 3 BioIntegrate, Lawrenceville, GA 30043, USA; 4 South Texas Orthopaedic Research Institute (STORI Inc.), Laredo, TX 78045, USA; anishpotty@gmail.com; 5 Northern Railway Central Hospital, New Delhi, Delhi 110025, India; drsuryaprakash5@gmail.com

Knee osteoarthritis (OA) is the most documented form of OA and is accountable for about 80% of total OA cases worldwide [1]. Lately, the incidence of knee OA has continued to rise, with no signs of slowing down [1]. Its etiology involves inflammation of the synovial tissue and degeneration of the articular cartilage, resulting in unbearable pain and functional impairment [2,3]. Conventionally, knee OA is managed by utilizing pharmacological agents, including non-steroidal anti-inflammatory drugs (NSAIDs), corticosteroids, viscosupplementation, and narcotics; non-pharmacological modes, such as activity modification, weight loss, diet change, and physical therapy; and surgery (especially in advanced stages of knee OA) when traditional treatment modalities have been ineffective [2,3]. These aforementioned therapies have limitations and side effects, persistently intending to reduce pain rather than targeting the underlying pathophysiology [2,3].

Of late, clinicians have adopted the intra-articular injection of platelet-rich plasma (PRP) and hyaluronic acid (HA), either alone or in combination, for managing knee OA. Studies, including systematic reviews and meta-analyses, have reported that both PRP and HA, administered individually, result in improved outcomes, including reducing pain and the progression of OA; the post administration outcomes accomplished by PRP were better compared to HA [4,5]. Additionally, studies have suggested that a combination of PRP and HA may provide synergistic effects, thereby relieving pain, improving function, and slowing the progression of knee OA [6]. However, the number of studies comparing the efficacy of a combination of PRP and HA with PRP alone are limited. 

In this editorial, we focused on a recently published prospective clinical study by Ciapini et al. [7], titled “Is the Combination of Platelet-Rich Plasma and Hyaluronic Acid the Best Injective Treatment for Grade II-III Knee Osteoarthritis? A Prospective Study”. In this single-center, double-blinded, prospective study, the authors investigated the efficacy of a combination of PRP and HA with PRP or HA alone in terms of functional recovery and pain control. The inclusion criteria included patients between 30–80 years old with radiographic evidence of Grade II or III (on the Kellgren–Lawrence scale) knee OA, pain or functional limitations in activities of daily living (ADL), and an absence of clinical or imaging signs of articular instability. The exclusion criteria included known hypersensitivity to HA, pregnancy and lactation, body mass index (BMI) > 40, chronic administration of anti-coagulant drugs or history of coagulopathies, neoplastic lesions, and kidney failure. Sixty patients met the inclusion/exclusion criteria and were included in the study. These patients were randomly divided into three groups, with ten males and ten females in each group (i.e., twenty subjects/group). Group A, Group B, and Group C received intra-articular injections of HA (2 mL/40 mg HA, 1550 KDa), PRP (4 mL), and PRP+HA, respectively. The Visual Analogue Scale (VAS) and Western Ontario and McMaster Universities Arthritis Index (WOMAC) were recorded to assess the patient’s pain and overall clinical condition at baseline and at 3 and 6 months post injection, respectively. No adverse events were reported in either group throughout the duration of the study. PRP+HA showed a statistically significant (*p* < 0.05) improvement on the VAS compared to both PRP and HA alone. For the WOMAC, all three groups showed improvement in 3 months, but only the PRP+HA group showed continued improvement at 6 months. Though there were no statistical differences between PRP and PRP+HA, the treatment with PRP (Group B and C together) showed significant differences (*p* < 0.05) compared to HA alone at 6 months. This study was not without limitations, including a small cohort size and short follow-up. In addition, despite using a kit for the PRP formulation, no characterization of prepared/used PRP (platelet count and concentration compared to whole blood, presence or absence of WBC or RBC, use of activator, etc.) was reported.

In summary, despite the constraints, I applaud the efforts of the authors as this study positively adds to the current literature that the administration of PRP with HA is safe and potentially efficacious in patients with mild-to-moderate knee OA. The results from this study are in accordance with a recent systematic review and meta-analysis that reported significant improvements in pain and function up to 1 year with PRP+HA compared to PRP alone [8]. On the other hand, a more recent systematic review and meta-analysis concluded that PRP+HA was not superior to PRP alone [9]. Despite contrasting results, both systematic reviews and meta-analyses [8,9] share similar shortcomings, including a small number of selected studies and variability in the composition of PRP used in the included studies. Thus, more adequately powered, multi-center, double-blinded, prospective, randomized controlled trials involving a well-characterized PRP formulation with a longer follow-up are warranted to further determine the efficacy of PRP+HA compared to PRP alone in patients with knee OA. 

As of 1 October 2023, there are only two (one each) on-going clinical studies registered on clinicaltrials.gov and the Chinese Clinical Trial Register (ChiCTR) (search terms: “knee osteoarthritis” and “platelet-rich plasma” and ”hyaluronic acid”) comparing the efficacy of a combination of PRP and HA with PRP alone for the treatment of knee OA. These trials are summarized in Table 1.

## Figures and Tables

**Table 1:** On-going clinical trials registered on ClinicalTrials.gov and Chinese Clinical Trial Register (ChiCTR) till 1 October 2023 comparing the efficacy of PRP+HA vs. PRP alone for the treatment of knee osteoarthritis.

Study Identifier	Biologic	Study Phase; Estimated Enrollment (N)	Primary Outcome Measure(s)	Recruitment Status	Country
NCT02964143	Cellular Matrix of PRP+HA vs. HA alone vs. PRP alone	Not applicable; N = 306	Variation of the pain between baseline and month 6 (*pain will be evaluated using the WOMAC A subscale; each item of this subscale will be scored using a 100 mm VAS at baseline and Month 6. The total WOMAC A score will be reported as a summed score of this subscale.*). [Time Frame: 6 months]	Unknown	Switzerland
ChiCTR2100050974	PRP+HA vs. HA alone vs. PRP alone	Phase IV; N = 54	A change of WOMAC score at 6 months follow up compared with the score at baseline. [Time Frame: 6 months]	Not yet recruiting	China

PRP: Platelet-rich Plasma, HA: Hyaluronic Acid, VAS: Visual Analogue Scale, WOMAC: Western Ontario and McMaster Universities Arthritis Index.
